# The predictive value of the prognostic nutritional index for postoperative acute kidney injury in patients undergoing on-pump coronary bypass surgery

**DOI:** 10.1186/s13019-019-0898-7

**Published:** 2019-04-11

**Authors:** Ahmet Dolapoglu, Eyup Avci, Tuncay Kiris, Onursal Bugra

**Affiliations:** 1 Department of Cardiovascular Surgery, Medical School, Balikesir University Tip Fakultesi, 10145 Balikesir, Turkey; 2 Department of Cardiology, Medical School, Balikesir University, 10145 Balikesir, Turkey; 3 Department of Cardiology, Izmir Katip Celebi University, Ataturk Training and Research Hospital, Basın Sitesi, 35360 Izmir, Turkey

**Keywords:** Acute kidney injury, Coronary artery bypass surgery, Prognostic nutritional index

## Abstract

**Background:**

We aimed to investigate the predictive value of the prognostic nutritional index (PNI) regarding the development of acute kidney injury (AKI) after elective coronary artery bypass grafting (CABG).

**Methods:**

A total of 336 consecutive patients with normal serum creatinine levels undergoing CABG were enrolled in this retrospective study. AKI was defined as meeting Acute Kidney Injury Network (AKIN) criteria based on the occurrence of creatinine changes within the first 48 h after CABG surgery. The patients were grouped according to whether they developed AKI or not into an AKI (−) and an AKI (+) group.

**Results:**

AKI developed in 88 (26.2%) of all patients. The PNI was independently predictive of AKI (OR: 0.829, 95% CI: 0.783–0.877, *p* <  0.001). Moreover, C-reactive protein (CRP), a history of diabetes mellitus, and positive inotropric usage were independent risk factors for AKI in the multivariate logistic regression analysis. The area under the curve (AUC) of the multivariable model, including positive inotrope support, a history of diabetes mellitus, and CRP, was 0.693 (95% CI: 0.626–0.760, *p* <  0.001) in predicting AKIN. When the PNI was added to the multivariable model, the AUC was 0.819 (95% CI, 0.762–0.865, z = 3.777, difference *p* = 0.0002). Also, the addition of the PNI to the multivariable model was associated with a significant net reclassification improvement estimated at 88.2% (*p* <  0.001) and an integrated discrimination improvement of 0.22 (*p* <  0.001).

**Conclusions:**

Our study demonstrated that decreasing the PNI could be associated with the development of AKI after coronary artery bypass surgery.

## Background

Postoperative acute kidney injury (AKI) commonly arises in patients undergoing cardiac surgery and is associated with worse outcomes [1]. It increases postoperative mortality, morbidity, and the length of the hospital stay. The incidence of AKI following coronary artery bypass grafting (CABG) ranged from 12 to 48.5% in previously published data and operative mortality in these patient ranged from 40 to 80% [2, 3]. The use of cardiopulmonary bypass (CPB) significantly increased the development of postoperative AKI in CABG surgery compared with the off-pump technique [4].

There are some well-known independent risk factors for AKI following cardiac surgery, including older age, body mass index, the duration of the CPB, hypertension, reduced left ventricular ejection fraction, and impaired preoperative renal function [5]. Although multiple etiologic factors play a role in the pathogenesis, the exact mechanism of AKI after cardiac surgery is not completely understood [6]. The most prominent studies [7, 8] have especially focused on inflammatory reactions and ischemia-reperfusion injury in the development of AKI during cardiac procedures.

Albumin is a serum protein that is mainly responsible for plasma oncotic pressure. It is a good indicator of patients’ nutritional status. Additionally, it has anti-inflammatory and anti-oxidative properties, such as binding to various toxic agents and scavenging free radicals [9]. For these purposes, preoperative albumin level is becoming an important factor to reduce the risk of the hazardous effects of surgery. A preoperative reduced albumin level is associated with an increased risk of postoperative mortality and morbidity in cardiac surgical procedures [10]. Preoperative hypoalbuminemia has been shown to be a major risk factor for AKI in off-pump coronary bypass surgery, but there is no clear consensus regarding CABG surgery with CPB [11].

Lymphocytes are one of the subtypes of the white blood cell and play a fundamental role during inflammation. The immune response to surgical stress and CPB is also lymphocyte dependent along with other anti-inflammatory factors and as a result, a low count can be a predictor of poor survival in cardiac surgery. Previous studies [12] have shown that a low preoperative lymphocyte count is an independent risk factor for a worse postoperative outcome and a higher AKI after adult cardiac surgery.

The combination of the serum albumin and lymphocyte count mainly demonstrates a patient’s immunonutritional status and reflects the prognostic nutritional index (PNI). The PNI is widely used to assess the prognosis for patients with cancer, liver cirrhosis, and chronic renal failure [13, 14]. However, the predictive value of the PNI for postoperative AKI has not previously been examined for patients with normal serum creatinine levels who are undergoing CABG using CPB. Accordingly, the aim of this study was to investigate the association of the PNI with AKI in these patients.

## Methods

### Study design

Patients with normal serum creatinine levels who underwent elective on-pump CABG surgery between September 2015 and September 2018 were retrospectively evaluated. The inclusion criteria for the study was followed; patients who underwent elective on-pump CABG surgery. Patients were excluded if they had emergency surgery, a preoperative creatinine level of above 1.2 mg/dl, preoperative dialysis requirements, other concomitant surgical procedures such as valve replacement/repair, an active infection or malignancy, and preoperative proteinuria. Demographic and clinical characteristics, surgical details, and postoperative outcomes were obtained from the patients’ charts. The study protocol was approved by the ethics committee of our hospital.

### Blood sampling

Upon presentation, venous blood samples were obtained from all the patients. Albumin and other biochemical markers were measured. The levels of serum creatinine (sCr) were measured at the baseline (before coronary artery bypass surgery) and within the first 48 h following the CABG procedure. The hematologic parameters were measured using an automated hematology analyzer system (Abbott Cell-Dyn 3700; Abbott Laboratories, Abbott Park, Illinois). Absolute cell counts were used to perform the subsequent analyses.

### Coronary artery bypass surgery procedure

All CABG surgeries were performed under general anesthesia with standard median sternotomy. Cardiopulmonary bypass was used in all operations with cross-clamped aorta under cardioplegic arrest and moderate hypothermia. Multidose cold blood cardioplegia were administered intermittently through the aortic root in all patients and retrogradely through the coronary sinus for myocardial protection. CABG was performed using conventional techniques, and complete revascularization was achieved in all the patients. After surgery, the patients were transferred to the intensive care unit. The patients were extubated when they breathed spontaneously, achieved adequate blood gases, and had stable hemodynamics.

### Diagnosis of AKI

AKI was defined according to the AKIN criteria. Postoperative AKI was staged according to the AKIN criteria for changes in Cr within 48 h of surgery [15]. Urine output was not used since data on urine output were not adequately recorded in all patients and may have been affected by diuretic use. AKI (+) was defined as an increase in serum creatinine of at least 0.3 mg/dl or 150–200% (1.5- to 2-fold) from the baseline. All AKI stages (1–2-3) were included in the AKI (+) group.

### Definition of the PNI

We calculated the PNI using the following formula: PNI = serum albumin levels (g/dl) × 10 + total lymphocyte count (permm3) × 0.005, as proposed by Onodera et al. [16]. Low PNI (*n* = 110) and high PNI (*n* = 226) groups were defined as patients having values in the third tertile (< 46.50) and higher 2 tertiles (≥ 46.50), respectively.

### Statistical analysis

Continuous variables were presented as mean values (standard deviation [SD]) or medians with ranges, and the categorical variables were expressed as percentages. The variables were compared using a 2-tailed student t test for the continuous variables of normal distribution or the Mann-Whitney U test for the continuous variables of non-normal distribution. A w2 test was used for the categorical variables. The effects of the various variables on AKI were calculated by univariate regression analysis. In these analyses, the variables with unadjusted *p* < .1 were identified as confounding factors and included in the multivariate regression analyses to determine the independent predictors of AKI. The predictive values of albumin, the PNI, and the lymphocytes were estimated by the areas under the receiver operating characteristic curve. We used the DeLong test to compare the area under the curve (AUC) with each of these parameters [17]. Moreover, the increased discriminative value of the PNI was also estimated using net reclassification improvement (NRI) and integrated discrimination improvement [18]. All the statistical tests were 2-tailed, and a *p* < .05 value was considered significant. All the analyses were carried out using SPSS version 15 (SPSS, Inc., Chicago, Illinois).

## Results

The baseline characteristics of the study groups are provided in Table 1. AKI developed in 88 (26.2%) of all patients. The patients in the AKI group were older than those without AKI (66.5 ± 7 vs. 63.3 ± 9, *p* = 0.003). Compared with the AKI (−) patients, a history of diabetes mellitus and chronic obstructive pulmonary disease (COPD) was more frequent in the AKI (+) patients. Moreover, the use of positive inotropes and diuretics was higher in patients with AKI than those without AKI (Table 1). The rate of mortality for AKI (+) patients was higher than for AKI (−) patients (13% vs. 1%, *p* <  0.001).

**Table 1:** Baseline characteristics of the study population

Variable	AKI(−) (*n* = 248)	AKI (+) (*n* = 88)	*P*-value
Age (years)	63.3 ± 9	66.5 ± 7	0.003
Female n (%)	59 (24)	25 (28)	0.390
History of COPD n (%)	21 (9)	15 (17)	0.025
Hypertension n (%)	57 (23)	25 (28)	0.309
Diabetes mellitus n (%)	73 (29)	46 (52)	< 0.001
Hyperlipidemia n (%)	45 (18)	24 (27)	0.069
Current smoking n (%)	56 (23)	20 (23)	0.977
IABP usage n (%)	5 (2)	5 (6)	0.082
Positive inotrope usage n (%)	44 (18)	33 (38)	< 0.001
Preoperative diuretic usage n (%)	32 (13)	22 (26)	0.008
Mortality	2 (1)	11 (13)	< 0.001
Dialysis requirement(%)	0 (0)	7 (8)	< 0.001

Abbreviations: *AKIN* acute kidney injury, *IABP* intraaortic baloon pump, *COPD* chronic obstructive pulmonary disease

The laboratory variables of the groups are shown in Table 2. The lymphocyte counts and serum albumin levels were significantly lower in AKI (+) patients than in AKI (−) patients (1.9 ± 0.8 vs. 2.2 ± 0.6, *p* <  0.001; 3.4 ± 0.5 vs. 3.9 ± 0.4, < 0.001). The patients in the AKI (+) group had higher levels of sCr and C-reactive protein (CRP) than the patients in the AKI (−) group. Moreover, the hemoglobin level and PNI were lower in AKI (+) patients compared with those without AKI. In contrast, the glomerular filtration rate (GFR) was lower in AKI (+) patients than in AKI (−) patients (Table 2).

**Table 2:** The laboratory findings of study population

Variable	AKI (−) (*n* = 248)	AKI (+) (*n* = 88)	*P* value
BMI ((kg/m^2^))	23 ± 3	23 ± 2	0.960
SCr^a^_adm_ (mg/dl)	0.87 (0.77–0.97)	0.95 (0.79–1.10)	0.045
eGFR (mL/minute/1.73 m^2^)	84.8 ± 18.4	78.4 ± 24.2	0.010
Serum albumin (mg/dl)	3.9 ± 0.4	3.4 ± 0.5	< 0.001
Lymphocyte count (× 10^3^/μL)	2.2 ± 0.6	1.9 ± 0.8	< 0.001
WBC (× 10^3^/μL)	8.61 ± 1.95	8.44 ± 1.89	0.466
Hemoglobin (g/dl)	13.0 ± 1.5	12.1 ± 1.6	< 0.001
LVEF (%)	50 ± 6	49 ± 7	0.283
CRP^a^(mg/dl)	3 (2–5)	4 (3–7)	0.017
CPB time (min)	60 ± 11	61 ± 11	0.388
X-Clamp time (min)	40 ± 10	41 ± 9	0.538
PNI	50.7 ± 5	43.7 ± 7	< 0.001

Abbreviations: *AKIN* acute kidney injury, *LVEF* left ventricular ejection fraction, *SCr* serum creatinine at admission, *eGFR* estimated glomerular filtration rate, *WBC* white blood cell, *BMI* body mass index, *CRP* C-reactive protein, *PNI* prognostic nutritional index, *CPB* cardiopulmonary bypass

^a^Comparison was made using Mann-Whitney *U* test at *P* < 0.05, and these values were described by median with inter-quartile range (25th and 75th percentile)

The independent predictors for AKI identified using the multivariate logistic regression analysis are presented in Table 3. The PNI was independently predictive for AKI (OR: 0.829, 95% CI: 0.783–0.877, *p* <  0.001, Table 3). In addition, CRP, a history of diabetes mellitus, and positive inotropic usage were independent risk factors for AKI in the multivariate logistic regression analysis.

**Table 3:** Univariate and Multivariate logistic regression analysis for AKI

	Univariate		Multivariate	
Variables	OR (95% CI)	*P*-value	OR (95% CI)	*P*-value
Age (year)	1.047 (1.016–1.079)	0.003		
Lymphocyte count^a^	0.389 (0.256–0.591)	< 0.001		
Albumin levels^a^	0.135 (0.075–0.242)	< 0.001		
PNI	0.808 (0.767–0.852)	< 0.001	0.829 (0.783–0.877)	< 0.001
Dyslipidemia	1.692 (0.957–2.990)	0.070		
Hemoglobine levels (mg/dl)	0.707 (0.599–0.834)	< 0.001		
Admission creatinine levels (mg/dl)^a^	8.307 (2.127–32.450)	0.002		
eGFR (mL/minute/1.73 m^2^)	0.983 (0.970–0.996)	0.011		
CRP	0.932 (0.910–0.954)	< 0.001	1.137 (1.006–1.286)	0.040
Preoerative diüretic usage	2.250 (1.224–4.136)	0.009		
Positive inotropic usage	2.782 (1.620–4.777)	< 0.001	2.171 (1.058–4.458)	0.035
IABP	2.928 (0.827–10.367)	0.096		
COPD	2.221 (1.089–4.532)	0.028		
Diabetes Mellitus	2.626 (1.593–4.327)	< 0.001	2.448 (1.313–4.563)	0.005

Abbreviations: *AKI* acute kidney ıinjury, *IABP* intraaortic baloon pump, *eGFR* estimated glomerular filtration rate, *COPD* chronic obstructive pulmonary disease, *PNI* prognostic nutritional index

^a^These parameters are not entered to the model in order to prevent multicollinearity

**Fig. 1:**
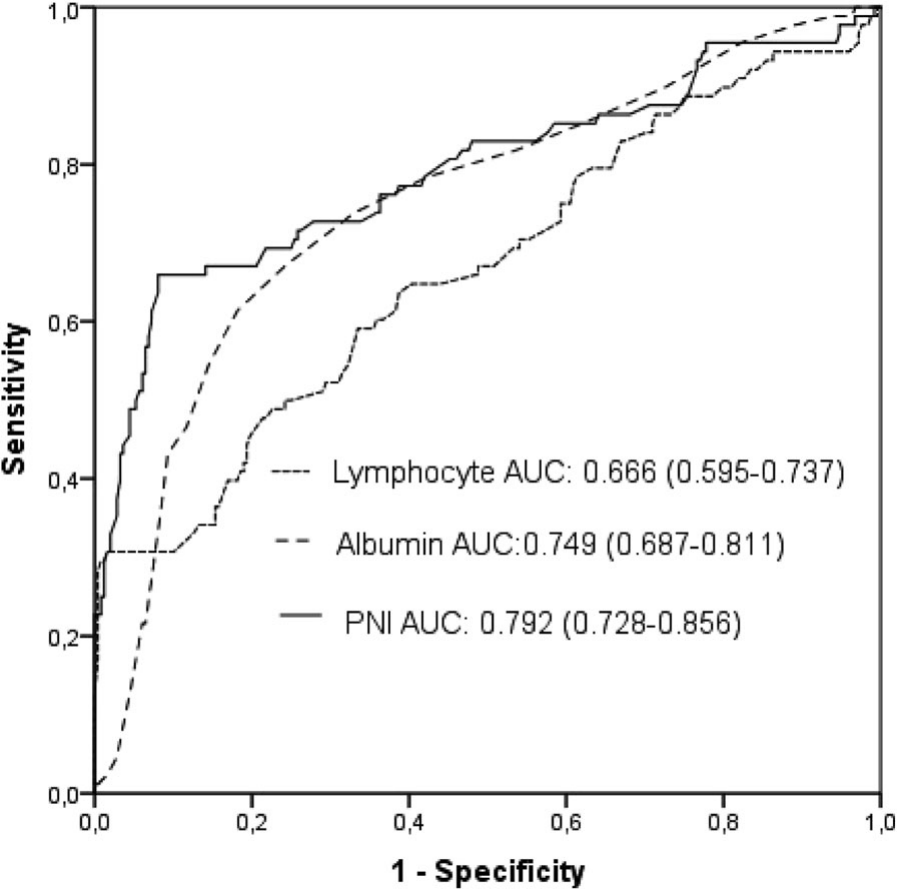
Receiver operating characteristic (ROC) curves for the albumin, lymphocyte counts, and prognostic nutritional index (PNI) for predicting AKI

The AUC for the PNI to predict AKI was 0.792 (95% CI: 0.728–0.856, *p* <  0.001, Fig. 1). Compared to both albumin and the lymphocytes, the PNI offered good accuracy in predicting AKI (PNI vs. albumin; AUC: 0.792 vs. 0.749, z = 2.016, *p* = 0.0438; PNI vs. lymphocyte; AUC: 0.792 vs. 0.666, z = 4.194, *p* <  0.001).

**Fig. 2:**
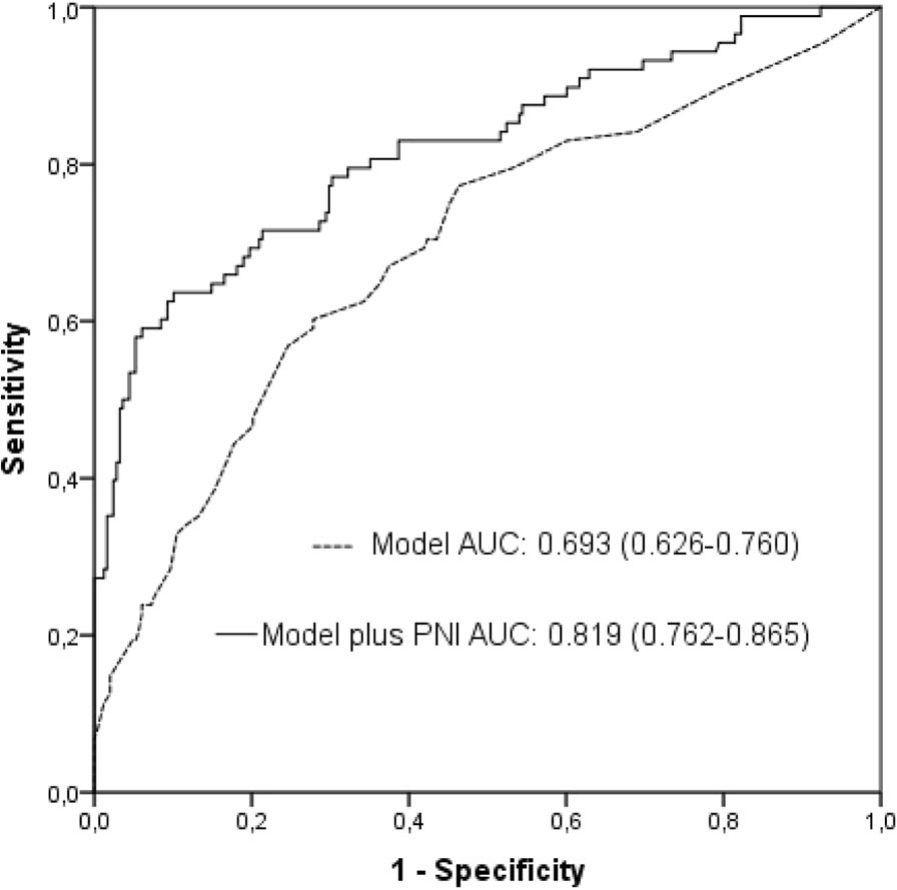
Receiver operating characteristic (ROC) curves for the multivariable model, and the multivariable model plus the prognostic nutritional index (PNI) for predicting AKI

For the development of AKI, the AUC of a multivariable model, including positive inotrope support, a history of diabetes mellitus, and CRP, was 0.693 (95% CI: 0.626–0.760, *p* <  0.001). When the PNI was added to a multivariable model, the AUC was 0.819 (95% CI: 0.762–0.865, z = 3.777, difference *p* = 0.0002, Fig. 2). Moreover, the addition of the PNI to a multivariable model was associated with a significant net reclassification improvement estimated at 88.2% (*p* <  0.001) and an integrated discrimination improvement of 0.22 (*p* <  0.001).

In sub-group analysis, the rate of AKI was higher in low PNI group compared with high PNI group (54% vs 13%, p < 0.001). Also, low PNI group had higher mortality rate than high PNI group (10% vs 3%, *p* = 0.001).

## Discussion

This study demonstrated that the PNI is an independent predictor of AKI in patients undergoing on-pump CABG surgery. To the best of our knowledge, this is the first study to investigate this relationship in these patients.

Postoperative AKI is an important clinical problem for patients undergoing coronary bypass surgery and the development of this complication is associated with an increased risk of mortality, morbidity, and medical costs [19]. The risk of death associated with AKI also remains high during long-term follow-up with these patients [4]. There is no specific treatment for postoperative acute kidney injury. Therefore, before the development of AKI, it could be a good strategy to predict and minimize the risk of occurrence.

The pathogenesis of the postoperative AKI is the consequence of multiple kidney aggressions occurring during the preoperative, intraoperative, and postoperative periods. There are various mechanisms responsible for the development of AKI in cardiac procedures, such as inflammatory reactions, ischemia-reperfusion injury, hemolysis, exposing nephrotoxic agents, and oxidative stress [20].

Postoperative AKI is significantly more common with CABG surgery using CPB than with the off-pump technique [21, 22]. Inflammation is becoming a more important factor for kidney injury when using CPB because it initiates a more prominent inflammatory response due to the contact of blood components with the artificial surface of the circuit. CRP is a representative marker for inflamation and several clinical settings have shown that a high CRP level is a predictor of AKI and mortality [23, 24]. Furthermore, Han SS et al. [25] have reported that an elevated preoperative CRP level predicts AKI after CABG surgery. Similarly, CRP has been found to be a predictor of postoperative AKI in our study.

Albumin is a serum protein that is a good indicator of a patient’s nutritional condition. It makes up the majority of the serum total protein and is mainly responsible for the serum osmotic presure. Besides its oncotic characteristics, albumin also has antioxidant and anti-inflammatory properties in scavenging and limiting the production of reactive oxygen radicals [26]. It has been shown that a reduced albumin level is associated with contrast-induced nephropathy, a common and important potential complication that occurs after angiography [27]. Hypoalbuminemia, especially in the postoperative period, has been shown to be an independent risk factor for a worse postoperative outcome in patients undergoing cardiac surgery [28]. The association between the preoperative albumin level and kidney injury has been investigated in coronary bypass surgery, mostly regarding the use of the off-pump technique, and preoperative hypoalbuminemia has been shown to be a major risk factor for AKI in off-pump CABG surgery [11, 29]. We have found only one study evaluating this association with the on-pump technique in the literature. Findik et al. [30] showed that a low preoperative albumin level was associated with an increased risk of renal failure in on-pump CABG surgery. Similarly, we found that a low preoperative albumin level was a predictor of postoperative AKI in the present study.

Lymphocytes are an important part of the immune system and the prognostic role of the lymphocyte count has been investigated in cardiac surgeries before [31]. Lymphocytes are an important part of the immune system and the prognostic role of the lymphocyte count has been previously investigated in coronary artery disease, myocardial infarction, and cardiovascular diseases [32–34]. Lymphopenia is a significant predictor of mortality in patients who underwent CABG surgery [35]. Aghdaii et al. [12] reported that a low preoperative lymphocyte count was associated with an increased risk of postoperative renal failure in cardiac operations. In our study, we discovered the relationship of the preoperative lymphocyte count to AKI.

There are some well-known preoperative, intra-operative, and postoperative risk factors for AKI during cardiac surgery, including diabetes, hypertension, obesity, older age, an elevated preoperative creatinine level, CPB time, and the use of postoperative inotropic [5]. As in previously reported data, we found that CPR, diabetes, and the use of inotropic agents were predictors of postoperative AKI in our study.

In clinical practice, the serum albumin level and lymphocyte count have been combinable and used for the PNI [16]. The PNI was originally designed to assess immunonutritional status. This risk index was widely used, especially for patients with cancer, malnutrition, and systemic inflammation, and for evaluations of surgical risk in gastrointestinal operations [36, 37]. Various studies have reported that a lower PNI level was significantly associated with higher mortality in patients with cardiovascular diseases, including myocardial infarction and pulmonary embolism [38]. The PNI was first examined in terms of coronary bypass surgery by Keskin et al., [39] and they found that a low preoperative PNI level is an independent prognostic factor for mortality in these patients. However, no previous studies have investigated the association between the preoperative PNI and the development of kidney injury among patients undergoing on-pump CABG surgery. In the present study, we found that a reduced PNI level was significantly associated with postoperative kidney injury in these patients.

Patients’ immunonutritional status can easily be revealed by using the PNI before surgical procedures. According to our results, the PNI might be considered a stronger anti-inflammatory factor than albumin or lymphocyte alone. A low PNI level may reflect the patient’s poor nutritional status before an operation and this may lead to a decay in intravascular osmotic pressure that is mainly created by albumin. Additionally, reduced preoperative PNI levels may indicate a decrease in the body’s immune response against acute distress, which is mainly aroused by surgery and the deterioration of intravascular osmotic pressure. It is probable that for these two reasons, postoperative AKI might be more commonly seen in patients with a low PNI who are undergoing an on-pump CABG procedure.

The present study had several limitations. We lacked detailed information about the patients’ postoperative hemodynamic conditions, which are known to affect the incidence of AKI development. This was a single-center, retrospective, and observational study. We lacked detailed information about the patients’ postoperative hemodynamic conditions, which are known to affect the incidence of AKI development. We didn’t separate the AKIN stage levels in our study due to the small proportion of patients in AKIN stage 2 and 3. For these reasons, we didn’t exactly estimate the association between the PNI and the severity of the AKI. Additionally, the PNI level might be influenced by hormonal changes, such as in serum catecholamine and cortisol; however, we could not measure these hormones in the present study. Also, frailty status which is associated with PNI is an another indicator of preoperative status. Moreover, the overall frailty status of a patient may be correlated renal functional reserve, and therefore it may constitute an independent risk factor for AKI in patients underwent CABG. However, frailty status was not measured in our study.

## Conclusions

The PNI score may be considered as a clinical element and indicator of AKI in these patients. This score may be used routinely to improve the identification of patients at higher risk for AKI before surgery.

## Abbreviations

AKI: Acute kidney injury
AKIN: Acute Kidney Injury Network
AUC: Area under the curve
BMI: Body mass index
CABG: Coronary artery bypass grafting
CI: Confidence interval
COPD: Chronic obstructive pulmonary disease
CPB: Cardiopulmonary bypass
CRP: C-reactive protein
GFR: Glomerular filtration rate
IABP: Intra-aortic balloon pump
LVEF: Left ventricle ejection fraction
NRI: Net reclassification improvement
PNI: Prognostic nutritional index
sCr: Serum creatinine
SD: Standard deviation
WBC: White blood cell
